# SRC-3, a Steroid Receptor Coactivator: Implication in Cancer

**DOI:** 10.3390/ijms22094760

**Published:** 2021-04-30

**Authors:** Licen Li, Chu-Xia Deng, Qiang Chen

**Affiliations:** 1 Cancer Centre, Faculty of Health Sciences, University of Macau, Macau, China; yb77636@connect.um.edu.mo (L.L.); cxdeng@um.edu.mo (C.-X.D.); 2 Centre for Precision Medicine Research and Training, Faculty of Health Sciences, University of Macau, Macau, China

**Keywords:** SRC-3, AIB1, coactivator, cancer

## Abstract

Steroid receptor coactivator-3 (SRC-3), also known as amplified in breast cancer 1 (AIB1), is a member of the SRC family. SRC-3 regulates not only the transcriptional activity of nuclear receptors but also many other transcription factors. Besides the essential role of SRC-3 in physiological functions, it also acts as an oncogene to promote multiple aspects of cancer. This review updates the important progress of SRC-3 in carcinogenesis and summarizes its mode of action, which provides clues for cancer therapy.

## 1. Introduction

Back to the early 1970s, it was discovered that non-histone proteins interact with DNA-binding nuclear receptors (NRs) to form complexes and support their functions [1]. These proteins are now known as NR coregulators, including coactivators and corepressors, to modulate gene expression in mammalian cells. The coactivator is a type of transcriptional coregulator that accomplish reactions required for activation of transcription. p160/steroid receptor coactivators (SRC) are about 160 kDa in size and consist of three members including SRC-1 (NCOA1), SRC-2 (NCOA2/TIF-2/GRIP-1) and SRC-3 (NCOA3/AIB1/ACTR/RAC-3/pCIP/TRAM-1) [2]. SRC-1 was the first NR coactivator identified by Bert W. O’Malley’s laboratory in 1995 [3]. A year later, SRC-2 was discovered as the second member of the SRC family [4]. Soon after, SRC-3 was identified on the long arm of chromosome 20 (20q12-13) known to be amplified in breast cancer and termed AIB1 (Amplified in breast cancer 1) [5,6]. SRCs were initially recognized as promoting the transcriptional activity of steroid hormone receptors, and then it was discovered that they can also modulate the activity of multiple other transcription factors.

The SRC-3 gene was first cloned from BT-474, a breast cancer cell line that contains high copy of chromosome 20 [5], five groups subsequently characterized SRC-3 as a coactivator, belonging to SRC family (AIB1, ACTR, RAC-3, pCIP, TRAM-1) [6,7,8,9,10]. In the past two decades, numerous studies have demonstrated that SRC-3 plays crucial roles in its physiology [11]. SRC-3 not only regulates body development but also maintains homeostasis. Deletion of the SRC-3 gene in mice caused growth retardation, dwarfism, abnormal mammary gland development and reproductive function [12]. Loss of SRC-3 also improved mitochondrial function via acetyltransferase GCN5-mediated peroxisome proliferator-activated receptor γ coactivator 1α (PGC-1α) activity, thereby increasing basal metabolism in BAT and preventing obesity [13]. In addition, SRC-3 plays important roles in innate immunity. SRC-3 null mice exhibited severe endotoxin-induced inflammatory response through enhancing pro-inflammatory cytokine mRNA translation [14], and were more susceptible to bacterial infections [15]. Besides its physiological effects, SRC-3 is best known for its oncogenic functions. In this review, we will mainly highlight the important progress related to cancer in past decade.

## 2. SRC-3 Structure, Isoform and Post-Translational Modification (PTM)

**Figure 1:**
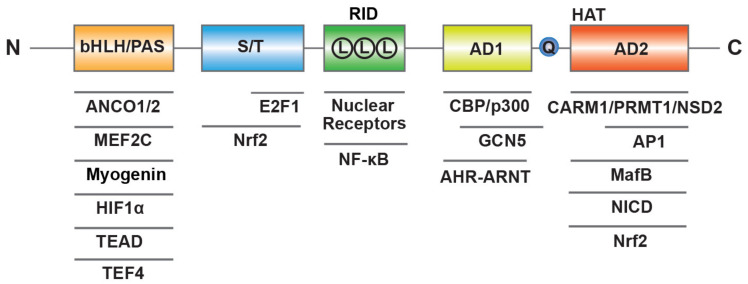
Molecular structure of human SRC-3. SRC-3 contains three structural and functional regions such as basic–loop–helix (bHLH) and Per/ARNT/Sim (PAS) domain, receptor-interaction domain (RID) with three LXXLL (L means LXXLL) motifs, transcriptional activation domains (AD1 and AD2) with a polyglutamate sequence (Q) and histone acetyltransferase (HAT) activity. SRC-3 interacts with multiple transcription factors and coregulators.

SRC-3 has a similar structure to the other members of SRC family, including three structural and functional regions (Figure 1) [16]. At the N-terminus, the basic–loop–helix (bHLH) and Per/ARNT/Sim (PAS) domain is the well-conserved region through SRCs, which is essential for a member of protein-protein interactions and nuclear localization. The central region is regarded as receptor-interaction domain (RID) and contains three LXXLL (L, Leucine; X, any amino acid) motifs that contribute to interactions with ligand-dependent NRs. At the C-terminus, there are two transcriptional activation domains (AD1 and AD2) as well as a polyglutamate sequence (polyQ) between them. In addition to exhibiting histone acetyltransferase (HAT) activity between the two AD regions, the AD1 and AD2 can interact with HAT (CBP/p300) or histone methyltransferases (CARM1, PRMT1 and NSD2), respectively, and then promote chromatin remodeling. In summary, these structure elements of SRC-3 provide a place where allow transcription factors interact with other coregulators to regulate gene expression.

Thus far, it has been reported that there are two splice variants of SRC-3, such as SRC-3Δ3 and SRC-3Δ4. SRC-3Δ3, an SRC-3 splice isoform with a deletion of exon 3, was determined to lack the N-terminal bHLH and PAS motif [17]. SRC-3Δ3 was more potent than full-length SRC-3 in enhancing estrogen receptor (ER) and progesterone receptor (PR) signaling, and its overexpression was found in breast cancer specimens [17,18]. These findings suggest that this isoform may play a role in pathogenesis of breast cancer. Based on SRC-3Δ3 transgenic mice, overexpression of this isoform promoted mammary epithelial cell growth, and eventually resulted in mammary hyperplasia and adenocarcinoma [19,20]. Similarly, SRC-3Δ4 is another splicing isoform of SRC-3 with a deletion of exon 4, which is predominantly localized in cytoplasm due to lack of nuclear localization sequence (NLS). However, SRC-3Δ4 could enter the nucleus to promote ER signaling by a non-canonical nuclear import pathway [17,21]. SRC-3Δ4 also worked as a signaling adaptor that mediates the interaction with epidermal growth factor receptor (EGFR) and focal adhesion kinase (FAK), thus promoting cell migration in an FAK-dependent manner. This was because p21-activted kinase 1 (PAK1) induced phosphorylation of SRC-3Δ4 to promote its membrane localization, thus bridging the interaction [22]. These findings imply that SRC-3 isoforms have more effective and distinct functions to regulate the development of cancer.

The function and level of SRC-3 are regulated by diverse PTMs including phosphorylation, methylation, acetylation, SUMOylation, ubiquitination and many other modifications [23]. Studying the PTM of SRC-3 is essential for understanding how to regulate the activity and level of SRC-3. Previous reviews summarized the modified residues, the responsible modifying proteins and the relative functions of SRC-3’s PTM [24,25]. Here, we update some new findings related to phosphorylation of SRC-3. SRC-3 has multiple phosphorylation sites including seven Serine/Threonine (T24, S505, S543, S601, S857, S860 and S867) phosphorylation sites [26] and one Tyrosine site (Y1357) [27], which control its transcriptional activity, protein stability and subcellular localization. Human epidermal growth factor receptor 2 (HER-2) is amplified in about 20% of all breast cancers and is associated with endocrine resistance and poor prognosis. A recent study indicated that decreased HER-2 signaling inhibited SRC-3 phosphorylation at T24, S543, S857 and S860, and the enhanced HER-2 signaling cooperated with phosphorylated SRC-3 to promote breast cancer cell proliferation [28]. According to reports, the most common phosphorylation site of SRC-3 is S857 [29]. Recently, two signaling axes were found to regulate phosphorylation of SRC-3 at this site [30,31]. Dasgupta et al. performed a kinome-wide RNA interference-based screening to identify fuctose-2,6-bisphosphatase 4 (PFKFB4) that phosphorylates SRC-3 at S857 and promotes the intrinsic SRC-3 transcriptional activity [30]. PFKFB4 is an isoform of phosphofructokinase 2 that synthesizes fructose 2,6-bisphosphate (F2,6-BP), an important glycolysis-inducing metabolite. The phosphorylation of SRC3 in cells was induced by glucose in a PFKFBP4-dependent manner, leading to transcriptional reprogramming and aggressive metastatic tumor. This study provides evidence for insight into the metabolism of cancer cells and their preference for glycolysis [32]. Based on in vitro phosphorylation assay, Shrestha et al. indicated that MK2 was responsible for phosphorylation of SRC-3 at S857 and p38 is the upstream of MK2. Activation of the p38-MK2 signaling leaded to the nuclear translocation of SRC-3, which contributed to NF-kB activation and downstream target expression [31]. In conclusion, PTMs are critical molecular events, which change SRC-3 conformation and generate diverse SRC-3 properties by regulating its stability, localization and interacting partners, consequently exerting pivotal roles in regulating the functions of SRC-3. Unraveling the specific role of SRC-3’s PTM and the responsible proteins in carcinogenesis is potentially useful for anti-cancer therapy. 

## 3. Implication of SRC-3 in Cancer

**Figure 2:**
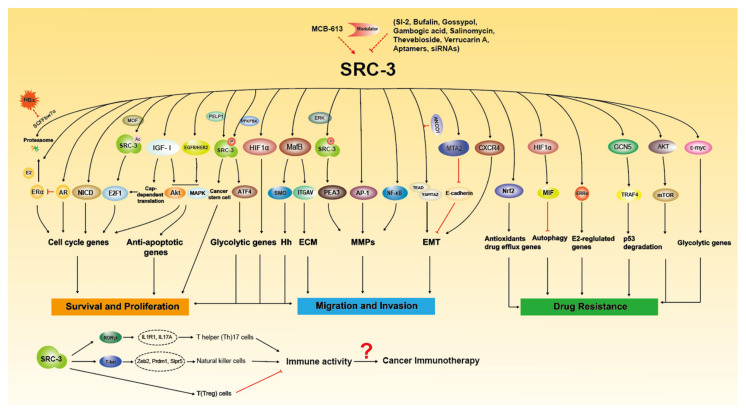
The molecular functions of SRC-3 in Tumorigenesis. SRC-3 promotes cancer growth, metastasis and drug resistance through multiple signaling pathways. SRC-3 modulators have been developed to regulate the expression and activity of SRC-3. SRC-3 affects the development of immune cells to regulate immune response, which suggest that SRC-3 may play certain role in cancer immunotherapy.

Since it was discovered in 1997 that SRC-3 is frequently amplified in breast cancer [6], extensive research has been conducted on the oncogenic role of SRC-3. SRC-3 amplification and overexpression have been found to be associated with tumor aggressiveness or poor prognosis in a number of clinical studies (Table 1), and SRC-3 promotes tumorigenesis and malignancy through multiple pathways (Figure 2). Based on previous reviews [24,33], we will summarize and update the important progress of SRC-3 relevant to cancers, including hormone-sensitive and non-hormone targeted cancers.

### 3.1. Hormone-Sensitive Cancers

Hormone-sensitive cancer is a type of cancer that depends on hormones for growth and/or survival, including breast cancer, ovarian cancer, prostate cancer, thyroid cancer and endometrial carcinoma. As a member of the SRC family, SRC-3 is required for the transcriptional activity of certain nuclear receptors, including ER, PR, androgen receptor (AR) and thyroid hormone receptor (TR), which not only control the development of related tissues, but also contribute to carcinogenesis of these tissues.

#### 3.1.1. Breast Cancer

Breast cancer has surpassed lung cancer as the leading cause of global cancer incidence in 2020 [76]. Among women, breast cancer has an estimated 2.26 million new cases and 680,000 deaths, accounting for one-quarter of cancer cases and one-sixth of cancer deaths [76]. Since SRC-3 was identified on the amplified chromosome in breast cancer, many clinical and basic studies have explored the functions of SRC-3 in the occurrence, progression and prognosis of breast cancer. Mouse mammary tumor models have shown that lack of SRC-3 impairs tumor formation [77,78], but overexpression of SRC-3 triggers malignancy of mammary epithelium [79,80,81], which indicate that SRC-3 is a crucial driver for mammary tumorigenesis.

##### Clinical Significance and the Regulation Mechanism of SRC-3 Expression

Initially, it was found that the SRC-3 gene was amplified in 10% of breast cancers [6]. Later studies showed that the amplification of this gene was observed in fewer breast cancers (1.6% to 4.8%) [34,35,36]. However, SRC-3 mRNA was found to be overexpressed in 31–64% of breast cancer [6,35], and 16% of breast cancer showed nuclear staining for SRC-3 protein [36]. Less than 10% of breast cancers overexpressing SRC-3 are related to gene amplification [6], indicating that transcriptional and/or posttranscriptional mechanisms are the main determinants for abnormal levels of SRC-3 in cancers. Early findings showed that steroid hormones and growth factors both can regulate SRC-3 expression at the mRNA level, but the molecular mechanisms were unclear [82]. According to the analysis of the SRC-3 promoter, it is found that E2F1, which is frequently overexpressed in cancers, enhanced SRC-3 expression by enhancing the promoter activity of SRC-3 [83]. ANCCA, as a coactivator for E2Fs, was another key mediator of SRC-3 overexpression [84]. Besides transcriptional regulation, the translation of SRC-3 mRNA could be inhibited by microRNA such as Mir-17-5p, which was down-regulated in breast cancer cell line [85]. In addition, the proteasome-mediated posttranslational protein turnover plays an important role in regulation of the SRC-3 levels, and some ubiquitin ligases, such as E6-AP [86], SCF^Fbw7α^ [87], CHIP [88], SPOP [89] and Cullin3 [90], have been found to participate in ubiquitination and degradation of SRC-3. In addition, REGγ, a proteasome activator, interacted with SRC-3 to promote the degradation of SRC-3 in a ubiquitin independent manner [91].

##### Hormone Dependent Signaling Pathway

The ERα and PR status of breast cancer are important biomarkers for breast cancer therapy and prognosis. AIB1 overexpression, especially nuclear expression, is associated with ERα and PR positivity, as well as tumor size [34,36]. SRC-3 is believed to play significant roles in ER positive breast cancer. SRC-3 interacts with ERα and enhances its activity by sequentially recruiting CBP/p300 and CARM1, and thereby facilitates the transcription of downstream genes (such as PR and cyclin D1) to promote cell proliferation [92,93,94,95,96,97]. SRC-3 also promotes epithelial-mesenchymal transition (EMT) through its interaction with ERα. The SRC-3-ERα complex directly bound to the ERα-binding site on the SNAI1 promoter, and increased the transcription of SNAI1, resulting in repression of E-cadherin expression in breast cancer [98]. However, AR weakened the interaction between SRC-3 and ERα, and thus decreased estrogen (E2)-mediated cyclin D1 expression. This suggests that targeting AR signaling may enhance the effectiveness of anti-estrogen adjuvant therapies [99]. A recent study has found that SRC-3 also functioned as a tether to generate enhancer-promoter contacts (EPCs) and induced the full transcriptional expression of the target gene *GREB1* in the presence of E2 [100]. In addition to regulating transcriptional activity, SRC-3 was also found to be required for E2-stimulated ERα degradation and turnover via the ubiquitin–proteosome machinery [101]. Thus, SRC-3 plays a dual role in regulating ERα activity.

##### Hormone Independent Signaling Pathway

According to mouse mammary tumor models, SRC-3 deficiency significantly impaired mammary tumorigenesis without alteration of the expression of E2 and progesterone-responsive genes [77,78], which suggests that SRC-3 also promotes breast malignant tumors through other signaling pathways. Loss of SRC-3 partially impaired the insulin-like growth factor I (IGF-I) signaling pathway and inhibited H-ras-driven tumor initiation [77]. IGF-I signaling controls protein synthesis by regulating some key translation mediators. A recent study has found that SRC-3 plays a regulatory role in polyribosome recruitment and the translational complex formation regardless of ERα, its knockdown suppresses a subset of IGF-I-stimulated translation of cancer-related mRNAs [102]. SRC-3 also controls EGFR and human epidermal growth factor receptor 2 (HER-2) phosphorylation to regulate cell proliferation [103]. In addition, SRC-3 is recruited to E2F target gene promoters through direct interaction with E2F1, thereby stimulating the transcription of G1/S cycle transition-related genes to promote E2-independent cell proliferation [104]. The acetylation of AIB1 by acetyltransferase MOF is required for its interaction with E2F1 [105]. As mentioned above, SRC-3 could promote its own expression through E2F1 [83], thus this positive feedback regulatory loop is believed to enhance the effect of SRC-3 on cell proliferation. Aside from cell proliferation, it is reported that SRC-3 interacted with ETS transcription factor PEA3 and enhanced the expression of target genes such as matrix metalloproteinase 2 (MMP2) and MMP9, thus promoting EMT and lung metastasis [106]. As a coactivator, SRC-3 also up-regulated MMP7 and MMP10 by mediating AP-1 activity to promote invasiveness [107]. According to comparative analysis of SRC-3 interactome, a recent study has shown that SRC-3 served as a transcriptional repressor by interacting with the chromatin remodeling factor MTA2, which inhibited the expression of E-cadherin to promote EMT and pro-metastatic phenotype in ER positive breast cancer [108]. SRC-3 also was required to maintain myoepithelial progenitor cells in ductal carcinoma in situ (DCIS) lesion via NOTCH and HER-2/HER-3 signaling molecules, thus increasing incidence of invasive breast cancer [109]. Recently, Dasgupta et al. demonstrated that the phosphorylation of SRC-3 induced by PFKFB4 increased its interaction with the transcription factor ATF4 to promote transcriptional reprogramming and aggressive metastatic cancer by stabilizing the recruitment of SRC-3 and ATF4 to downstream gene promoters [30].

##### Drug Resistance

Since the 1970s, tamoxifen has been considered as the first-line hormone therapy for ER-positive breast cancer. However, acquired resistance to tamoxifen has become a great challenge in breast cancer treatment. High levels of SRC-3 are associated with disease-free survival of patients treated with tamoxifen, which is indicative of tamoxifen resistance [37]. Furthermore, high SRC-3 expression in patients with high levels of HER-2 or other HERs was associated with higher risk of relapse and worse outcome on tamoxifen therapy [37,38]. Then, it was found that tamoxifen significantly increased SRC-3 expression independent of dose, which was positively correlated with HER-2 expression [39]. Based on tamoxifen-resistant cells, SRC-3 was found to be required for tamoxifen resistance, and the silence of AIB1 resulted in the restoration of tamoxifen sensitivity [110]. In addition, tamoxifen-resistant cells showed high levels of acetyltransferase GCN5 due to its reduced proteasomal proteolysis, thereby increasing SRC-3 expression to induce p53 degradation and tamoxifen resistance [111]. In terms of molecular mechanism, tumor necrosis factor receptor associated-factor 4 (TRAF4), a downstream gene of SRC-3, competed with p53 to interact with deubiquitinase HAUSP, and then induced p53 proteasomal degradation and resistance to cytotoxic agents [112]. SRC-3 also directly interacts with estrogen-related receptor α (ERRα) and increased endocrine resistance by controlling E2-regulated genes in a hormone-independent manner [113]. Autophagy plays a dual role in cancer progression, including pro- and antitumor effects [114]. As a coactivator, SRC-3 was recruited to the promoter of macrophage migration inhibitory factor (MIF) and promoted its expression through interaction with transcriptional factor hypoxia-inducible factor (HIF) α. MIF is a strong inhibitor of autophagic cell death, so the removal of AIB1 induced autophagic cell death and enhanced chemosensitivity by suppression of MIF [115]. Therefore, SRC-3 is a potential therapeutic target combined with hormone therapy or chemotherapy.

##### Triple Negative Breast Cancer (TNBC)

TNBC is a more aggressive type of breast cancer defined by the lack of ER, PR and HER-2 and has no established molecular targets for therapy [116]. High expression of SRC-3 is significantly associated with poor prognosis of TNBC patients [117], suggesting that SRC-3 also plays a role in TNBC. As the above mentioned, SRC-3 promotes the progression of breast cancer via ER independent signaling such as E2F1, IGF-I, PEA3, AP-1 and ATF4. SRC-3 was also characterized as a binding partner of cytoplasmic proline, glutamic acid and leucine-rich protein 1 (PELP1). Cytoplasmic PELP1 overexpression increased phosphorylation of SRC-3 and the expression of target gene independent of hormone stimulation, thereby promoting stem-like cells enrichment and E2-independent breast cancer progression [118]. The coactivators YAP/TAZ and target genes are elevated in TNBC [119]. TEADs are the primary transcription factors for the YAP/TAZ in the Hippo pathway. Recently, studies have found that SRC-3 formed the complex with TEAD and YAP/TAZ and resulted in transcriptional reprogramming including de-repression of transcription at 1q21.3 and activation of a genome-wide oncogenic transcription, then promoted the development and progression of TNBC [120,121]. In addition, one subtype of TNBC is driven by AR, called luminal androgen receptor (LAR) subtype, which is sensitive to AR antagonists [122]. A recent clinical study demonstrated that AR could be a potential target for this subtype [123]. As a coactivator of AR, SRC-3 may promote the progression of LAR subtype through AR signaling. Further study can focus on this perspective to find more beneficial effects in the treatment of TNBC. However, SRC-3 may have distinct effect on chemotherapy resistance in TNBC. According to a recent study [124], transcriptional signature of SRC-3 knockdown cells displayed a signature indicative of poor response to chemotherapy in TNBC patients, but the mechanism is unclear.

#### 3.1.2. Ovarian Cancer, Endometrial Carcinoma and Cervical Cancer

Estrogen and progesterone signaling are important in tumorigenesis of hormone-dependent tissues, so the enhancement of these signaling activation by SRC-3 might be an important step in the development and progression of ovarian cancer, endometrial carcinoma, and cervical cancer.

Besides breast cancer, AIB1 was also found to be overexpressed or amplified in ovarian cancer. Based on 122 ovarian tumors, amplification of the SRC-3 gene was observed in 7.4% of ovarian cancers, which was even higher than those detected in breast cancer (4.8%) [34]. Among 24 cases of sporadic ovarian cancer, 25% showed amplification of the SRC-3 gene, which was related to ER positivity and poor prognosis [41]. The expression of SRC-3 was also significantly associated with advanced ovarian cancers and platinum resistance [42,43]. In addition, polymorphisms of polyQ region within SRC-3 has been associated with prognosis of ovarian cancer, with a short polyQ genotype related to rapid recurrence [44]. These clinical data suggest a role for SRC3 in the progression of ovarian carcinoma. According to analysis of the expression correlation between SRC-3 and long non-coding RNA (lncRNA), TUG1 was positive correlated with SRC-3, showing a tumor-promoting effect [125], but the related molecular mechanism need to be further resolved.

Although there is no evidence showing the role of SRC-3 in the pathogenesis of endometrial carcinoma, several clinical studies have investigated the expression of SRC-3 in endometrial carcinoma and its effect on prognosis of this disease. SRC-3 overexpression at the mRNA level was observed in 17% of endometrial carcinoma, but no gene amplification was found in this cohort [40]. High mRNA level of SRC-3 was significantly associated with poor survival of patients, suggesting that SRC-3 could be considered as a predictor of prognosis of this disease [45]. In addition, according to immunohistochemistry (IHC), high SRC-3 protein expression was also related to poor prognosis and ER nuclear expression, indicating that augmented ER activity may cause endometrial hyperplasia and progression to malignancy [46]. In future studies, it is necessary to explore the correlation between SRC-3 and hormonal therapy of endometrial carcinoma and its related mechanisms. Cervical cancer is the fourth most common type of cancer among women worldwide [76]. High-risk human papillomaviruses (HPVs) are the major carcinogens for cervical cancer and are responsible for most of this disease [126]. However, ER signaling is associated with HPV infection and cervical cancer in many aspects, including pro-carcinogenic and anti-carcinogenic relationship [127]. Recent study found that SRC-3 was more frequently high expressed in cervical cancer (52.7%) compared to normal cervical tissues (30%), and high level of SRC-3 is significantly associated with aggressiveness and chemoradiotherapy resistance [47]. It suggests that SRC-3 may be considered as a potential target and predictor for the treatment of this disease.

#### 3.1.3. Prostate Cancer

Prostate cancer is the second most frequent cancer among men globally [76]. AR, a steroid receptor transcriptional factor, plays pivotal roles in all stages of prostate cancer [128]. It has been found that SRC-3 is a preferred coactivator for ligand-binding AR via LXXLL motif [129], and enhances AR-mediated transcriptional activity under androgen stimulation [130]. In prostate cancer patients, SRC-3 was found to be overexpressed and its overexpression was closely related to proliferation, metastasis and poor prognosis [48,49,50]. It indicates that SRC-3 has an important role in the progression of prostate cancer. According to transgenic adenocarcinoma of the mouse prostate (TRAMP) mice, global ablation of SRC-3 or its specific deletion in prostatic epithelial cells blocked prostate tumorigenesis, especially neuroendocrine tumor cells (NETCs) formation [131,132]. In terms of molecular mechanism, SRC-3 is necessary for cell survival, proliferation and migration regardless of AR. SRC-3 and AR not only can coordinately regulate the expression of cell cycle-related proteins [133], SRC-3 can also cooperate with AP-1 to induce the transcription of components involved in IGF/Akt pathway, thereby promoting cell survival and proliferation [49,134,135]. Moreover, SRC-3 affected the focal adhesion turnover via regulation of focal adhesion kinase activation as well as served as a coactivator of AP-1 and PEA3 to promote MMP-2 and MMP-13 transcription, thus enhancing prostate cancer cell migration and invasion [50].

Androgen deprivation therapy is the first-line treatment for advanced prostate cancer, however, most tumors will relapse and become hormone resistant, called castration-resistant prostate cancer (CRPC). Prostate cancer mainly relies on AR mutations, overexpression and changes of related cofactors to adapt to survival, thus becoming CRPC [128]. It has been shown that AR mutations found in prostate cancer patients were related to their affinity for SRC-3 [129], and SRC-3 promoted the development of prostate cancer through AR-dependent and -independent signaling. These suggest that SRC-3 may contribute to CRPC development. Compared with primary tumors, high expression of SRC-3 was observed in CRPC samples, and SRC-3 promoted the development of CRPC in PTEN mutant mice via Akt-mTOR signaling [136]. Therefore, disruption of SRC-3 is a potential strategy for inhibiting CRPC development caused by androgen deprivation therapy.

#### 3.1.4. Thyroid Cancer

Thyroid cancer is a rare type of cancer that affects the thyroid gland, and contains several types such as papillary, follicular, anaplastic and medullary types. It is one of the highest growing cancer diagnoses worldwide, especially in the female population. The etiology of thyroid cancer is not well understood [76]. Based on IHC staining, SRC-3 overexpression was detected in 60.2% of papillary thyroid carcinoma (PTC), and its up-regulation was positively correlated with lymph node metastasis [51]. In another cohort, positive SRC-3 expression was found in 54.6% of anaplastic thyroid carcinoma (ATC), an aggressive form of thyroid cancer, but SRC-3 levels in PTC and follicular thyroid carcinoma (FTC) were not significantly different from normal thyroid tissues [52]. Although the results are inconsistent, it suggests that SRC-3, which was identified as thyroid hormone receptor (TR) [7], may play a role in thyroid cancer. TRβ gene mutations results in resistance to thyroid hormone (RTH), and spontaneously induces the development of FTC. In TRβ mutant (TRβ^PV/PV^) mice model, SRC-3 was thought to regulate the thyroid and pituitary growth through two pathways, including regulation of TR activity as a co-regulator and IGF-1/PI3K/Akt/mTOR signaling [137]. Moreover, disruption of SRC-3 in TRβ^PV/PV^ mice significantly inhibited tumor growth and metastasis via inhibiting the expression of E2F1, Bcl-2 and vascular endothelial growth factor (VEGF) [138].

### 3.2. Non-Hormone Targeted Cancers

Besides steroid hormone receptors, SRC-3 also functions as a coactivator via other transcription factors. Therefore, SRC-3 is also associated with non-hormone targeted cancers, such as liver cancer, pancreatic cancer, lung cancer, gastric cancer, colorectal cancer, bladder cancer, etc.

#### 3.2.1. Liver Cancer

Primary liver cancer includes two major types, hepatocellular carcinoma (HCC) and intrahepatic cholangiocarcinoma (CCA), and is the third leading cause of cancer death worldwide [76]. In HCC patients, amplification of SRC-3 was more frequently detected in metastatic and recurrent HCC than in primary HCC [53]. Moreover, SRC-3 was found be overexpressed in 51.1–68% of HCC and its overexpression was highly associated with poor prognosis [54,55]. In addition to gene amplification, overexpression of SRC-3 can also be regulated in transcriptional and post-translational pathways. Majaz et al. found that GCN5 was up-regulated in HCC patients and increased the transcription of SRC-3 by cooperating with E2F1 [139]. It is well-known that hepatitis B virus (HBV) infection is one of main risk factors for HCC development [140]. We found that HBV X protein (HBx), a regulator of HBV replication, could interact with SRC-3 to disrupt the interaction between SRC-3 and E3 ligase Fbw7α, thus inhibiting Fbw7α-mediated ubiquitination and degradation of SRC-3 [141]. Up-regulation of SRC-3 in HCC implies that SRC-3 plays a role in the progression of HCC. Our study demonstrated that SRC-3 could promote cell proliferation via Akt activation and enhance cell invasiveness via the expression of MMP-9 mediated by NF-κB and AP-1 [55]. HBx also cooperated with SRC-3 to promote HCC cell invasiveness through NF-κB activation [141,142]. However, a recent study showed that SRC-3 inhibited HBV biosynthesis via activation of Akt signaling to impair hepatocyte nuclear factor 4α (HNF4α) nuclear translocation [143]. It suggests that there is negative feedback on HBx-SRC-3 axis.

Sorafenib is the first-line therapy for advanced HCC; however, sorafenib resistance has been considered as the major challenge in the therapy. Recently, it is found that SRC-3 was significantly up-regulated in sorafenib-resistant HCC xenografts, and played a crucial role in sorafenib resistance through regulation of the Warburg effect [144]. SRC-3 interacted with c-Myc, and then facilitated c-Myc recruitment to the promoters of glycolytic genes [144]. Another study also showed that overexpression of SRC-3 caused HCC cells to develop resistance to sorafenib [145]. These findings suggest that SRC-3 inhibition and sorafenib combination treatment might be a promising therapy for HCC.

Aside from HCC, we also found that SRC-3 was overexpressed in human CCA specimens and cell lines [56]. SRC-3 not only promoted the proliferation of CCA cells through Akt signaling activation, but also served as a coactivator of NF-E2-related factor 2 (Nrf2), a critical transcription factor of antioxidants and detoxification enzymes, to increase downstream gene expression, thereby resulting in CCA progression and resistance to chemotherapy [56].

#### 3.2.2. Pancreatic Cancer

Pancreatic cancer, also known as pancreatic adenocarcinoma, is one of the most aggressive cancers. It has been projected that pancreatic cancer will become the second leading cause of cancer death by 2030 [146]. Amplification of SRC-3 was observed in six of nine pancreatic cancer cell lines [147] and the expression of SRC-3 protein was found to be up-regulated in pancreatic adenocarcinoma and its precursor lesions [57]. Moreover, the frequency of SRC-3 overexpression in pancreatic adenocarcinomas with lymph node metastasis was significantly higher than that in tumors without metastasis [58]. These clinical studies propose a major role of SRC-3 in the progression of pancreatic cancer. Recently, we found that SRC-3 enhanced the proliferation and invasion of pancreatic cancer cells via Hedgehog (Hh) and extracellular matrix (ECM) signaling [148]. SRC-3 could function as a coactivator to enhance the activity of transcription factor MafB and induce the expression of upstream factors of Hh and ECM signaling, such as smoothened (SMO) and integrin αv (ITGAV), thereby promoting cell cycle progression and EMT. The overexpression of SRC-3 was correlated with low expression of E-cadherin in pancreatic cancer, which is consistent with our observation in cell lines [58]. Our finding also suggests that SMO could be potential therapeutic target for pancreatic cancer with SRC-3 high expression [148].

#### 3.2.3. Lung Cancer

Although the incidence of lung cancer is surpassed by female breast cancer in 2020, lung cancer is still the leading cause of cancer death worldwide [76]. Non-small-cell lung cancer (NSCLC) is the main type of lung cancer, accounting for about 85% of all lung cancers. According to 230 NSCLC patient cohorts, amplification and overexpression of SRC-3 were observed in 8.2% and 48.3%, respectively. Its overexpression was highly associated with poor prognosis and was independent of steroid hormone receptors such as ER, PR and AR [59]. In another NSCLC cohort, high level of SRC-3 was detected in 27% of NSCLC and lung cancer cell lines [60]. Down-regulation of SRC-3 significantly inhibited tumor cell growth and caused apoptosis [60]. Besides cell survival, SRC-3 also enhanced invasiveness of lung cancer cells via multiple pathways. C-X-C motif chemokine receptor 4 (CXCR4) plays an important role in the cell proliferation and metastasis of lung adenocarcinoma. SRC-3 overexpression could increase CXCR4 expression to promote metastasis [149]. In addition, the atypical MAPK ERK3 signaling induced SRC-3 phosphorylation at S857 through the interaction of SRC-3 and ERK3. The phosphorylation was required for interaction of SRC-3 with PEA3, which promoted the expression of MMP genes and invasive activity in lung cancer cells [150].

Tyrosine kinase inhibitors (TKI) against EGFR, such as Gefitinib, are used as targeted therapy for lung cancer with EGFR gene changes, but many tumors acquire TKI resistance after treatment. Cai et al. indicated that the level of SRC-3 was correlated with Gefitinib resistance in lung cancer and SRC-3 knockdown caused TKI-resistant lung cancer to be more sensitive to Gefitinib [60], which suggests SRC-3 may be a therapeutic target for lung cancer in combination with Gefitinib.

#### 3.2.4. Gastric and Colorectal Cancer

Colorectal cancer (CRC) and gastric cancer (GC) remain important cancers worldwide, and their incidence ranks third and fifth, respectively [76]. It has been reported that the overexpression and amplification of SRC-3 was detected in 35% and 10% of the 85 CRCs, respectively, and SRC-3 overexpression was highly correlated with advanced CRCs [61]. Besides clinical specimens, SRC-3 was also highly expressed in CRC cell lines. Disruption of SRC-3 not only inhibited the proliferation and metastasis of CRC cells, but also impaired colon carcinogenesis in colitis-associated cancer model [151]. Moreover, SRC-3 could directly bind to the Notch intracellular domain (NICD) and Mastermind-like1 (MAML1) to enhance Notch signaling, thereby promoting the development of CRC [151]. For GC patients, the amplification and overexpression of SRC-3 were found in different cohorts, and its amplification was significantly associated with metastasis and poor prognosis of GCs [62,63,64]. SRC-3 could exacerbate the malignancy of GC through regulating ErbB and Wnt/β-catenin pathways [63]. Collectively, SRC-3 represents a potential prognostic marker and therapeutic target for CRC and GC.

#### 3.2.5. Bladder Cancer

Bladder cancer is the sixth most common cancer among men in 2020. Urothelial carcinoma (UC), including lower tract (LTUC) and upper tract (UTUC), is the major type, which accounts for about 90% of all bladder cancers. In 163 primary UCs, the overexpression and amplification of SRC-3 were observed in 32.5% and 7.0%, respectively, and its overexpression was significantly associated with shortened patient survival [65]. In another cohort, 31.5% of bladder cancer patients showed a high level of SRC-3 [66]. In addition, it was also found that high levels of SRC-3 are negatively related with survival rates of UTUC. [67,68]. SRC-3 could regulate the expression of cell cycle proteins through E2F1 and Akt signaling and promote the proliferation of bladder cancer cells [66]. SRC-3 also directly interacted with HIF1α and enhanced glycolysis related genes, such as glut1 and pgk1, to induce metabolic reprogramming in UC [152]. Thus, SRC-3 could be an independent biomarker for poor prognosis of patients with UC, and an intriguing drug target for therapy.

#### 3.2.6. Other Cancers

Besides the above-mentioned cancers, SRC-3 was also found to be significantly associated with the prognosis of other cancers (Table 1), including female glioma [69,70], nasopharyngeal carcinoma (NPC) [71], esophageal squamous cell carcinoma (ESCC) [72,73,74] and bone cancer [75]. However, the mechanism by which SRC-3 regulates the progression of these cancers has not been further elucidated. Recent study presented that overexpression of SRC-3 was correlated with poor outcome of multiple myeloma patients [153]. SRC-3 interacted with histone methyltransferase NSD2 and enhanced its liquid–liquid phase separation, thereby altered the transcriptome to compromise the sensitivity of myeloma cells to bortezomib treatment [153]. Interestingly, SRC-3 could inhibit the formation of lymphoma as tumor suppressor. Loss of SRC-3 resulted in malignant B-cell lymphoma in mice upon aging through NF-κB activation [154]. However, a recent study showed that overexpression of SRC-3 was observed in B-cell Non-Hodgkin lymphoma specimens and cells, down-regulation of SRC-3 resulted in cell cycle arrest and apoptosis [155]. Therefore, the role of SRC-3 on cancer is still not fully understood, and the genes that drive cancer may determine the function of SRC-3 in tumorigenesis.

## 4. SRC-3 as Therapeutic Target

Since SRC-3 plays an important role in a variety of cancers, recent studies have explored how to target SRC-3 and reported that some drugs targeting SRC-3 are used in cancer treatment. Gambogic acid (GA), a main active ingredient in gamboge, exhibits effective anti-cancer activity in multiple cancer cells. Studies have reported that GA could reduce the expression of SRC-3 and inhibit the proliferation of cancer cells [155,156]. Cullin3, an E3 ligase involved in SRC-3 ubiquitination [90], was up-regulated under GA treatment, which may contribute to SRC-3 degradation [155]. These findings suggest that GA could be used as a potential inhibitor of SRC-3 for cancer therapy. Salinomycin, an antibiotic, was found to down-regulate the transcription of SRC-3 and increase the sensitivity of breast cancer cells to tamoxifen treatment, which indicates salinomycin may be developed as SRC-3 inhibitor for endocrine-resistant breast cancer [157]. However, it has not been examined whether GA and salinomycin have an effect on the expression of SRC-1 and SRC-2. Recently, it was discovered that thevebioside (THB), an active component from traditional Chinese medicine, specifically suppressed the expression of SRC-3 via ubiquitin–proteasome-mediated degradation, but not SRC-1 and SRC-2, thus inhibiting the proliferation of NSCLC cells [158].

To repurpose the chemicals for targeting SRC-3, a high throughput screening assay was performed according to PubChem bioassay bioinformatic resource. 2,2-bis-(Formyl-1,6,7-trihydroxy-5-isopropyl-3-methylnaphthalene (gossypol) was identified as directly binding to SRC-3 via its RID domain, and down-regulating the protein level of SRC-3 in multiple cancer cell lines through a proteasome-independent pathway [159]. In addition, the cardiac glycoside bufalin was found be as a potent small-molecule inhibitor for SRC-3 through protein degradation [160]. Bufalin not only inhibited tumor growth via p53-mediated senescence [117,160,161], but also improved the sensitivity of cell cells to HDAC inhibitors [162]. However, gossypol and bufalin also affected the expression of SRC-1 [159,160], indicating they are not specific inhibitors of SRC-3. In addition, the cardiotoxicity of bufalin limits its clinical application. Then, Verrucarin A has been determined to selectively promote the degradation of the SRC-3 protein to inhibit proliferation and migration of cancer cells but has little effect on SRC-1 and SRC-2 [163]. Since Verrucarin A has no direct interaction with SRC-3, it may affect the upstream pathway to regulate SRC-3 degradation [163], which cannot rule out the impact on other proteins. Recently, SI-2 was developed as a new and effective small-molecule inhibitor for SRC-3 [164]. SI-2 can reduce the protein level of SRC-3 as well as directly interact with SRC-3 to inhibit its transcriptional activities. Based on toxicity assay, no acute or chronic toxicity of SI-2 was observed in vivo. Therefore, SI-2 is considered as a highly promising inhibitor for SRC-3 and high potential candidate for developing an anti-cancer drug [52,118,153,164,165,166,167,168]. Besides identifying chemicals used to block the activity of SRC-3, recent studies have also developed new methods for targeting SRC-3. Since cancer cells heavily depend on SRCs to maintain homeostasis, excessive SRCs activation may be able to disrupt the critical homeostasis, leading to stress accumulation and cell death [169]. MCB-613 was discovered as an effective SRC stimulator. It caused hyper-activation of SRCs including SRC-3 and resulted in cancer cell death through uncontrolled ER stress and excessive ROS [169]. Therefore, super-activation of SRC-3 is a potential alternative strategy for cancer treatment. Aptamers are single-stranded oligonucleotides that bind to a specific target molecule. They are widely used for targeting molecules due to many advantages, such as high affinity and specificity, low toxicity and immunogenesis, rapid and reproducible synthesis, etc. [170]. Based on SELEX (Systematic evolution of Ligands by Exponential Enrichment), it was found that a DNA aptamer AY-3 interacted with SRC-3 and impaired the interaction between SRC-3 and p300 [171]. It suggests that AY-3 may inhibit the activity of SRC-3 and become a potential candidate for developing an SRC-3 inhibitor. In addition, to silence SRC-3 expression by RNAi technology in vivo, nanoparticles have been developed as carriers to deliver siRNAs against SRC-3 [172,173]. The findings present that nanoparticles are a promising system for effective delivery of siRNA to down-regulate SRC-3 expression and inhibit tumor growth. In short, as technology advances, more drugs and methods will be developed to inhibit SRC-3 activity and treat cancers.

## 5. Conclusions

In the past decades, basic and clinical research on SRC-3 has involved in various cancers. With the deepening of research, the roles of SRC-3 in tumorigenesis show diversity and complexity. In this review, we summarize the diverse functions of SRC-3 in tumorigenesis and the development of SRC-3 modulators. Although this information enhances our understanding of the implication of SRC-3 in cancer, there are still some questions that need to be explored in depth. As described above, the level of SRC-3 is significantly associated with the progression and drug resistance of cancers. Therefore, the level of SRC-3 in tumors is a potential biomarker for cancer diagnosis. Besides the conventional methods such as IHC and fluorescent in situ hybridization (FISH), the new techniques will be developed to measure the expression and amplification of the SRC-3 gene. A recent study invented a new method for monitoring SRC-3 levels in a breast cancer cell based on aptamer-functionalized nanomotors [174]. However, whether this method can be used to detect SRC-3 in tissues needs to be further clarified. In addition, it is found that SRC-3 is also involved in the development of immune cells. SRC-3 is not only critical for the maturation of effector T helper (Th)17 cells in a ROR_γ_t-dependent manner [175], but also the differentiation of suppressor regulatory T (Treg) cells [176]. SRC-3 also plays an important role in regulating the maturation of natural killer cells by promoting the transcriptional activity of T-bet [177]. These findings imply that SRC-3 may be a critical factor for cancer immunotherapy, which motivates further investigations in the future.

## Figures and Tables

**Table 1:** Gene amplification and expression of SRC-3 in cancer and clinical significance.

Cancer Type/Changes	Method	Frequency (n)	Molecular Association	Prognostic Association	Refs
Breast cancer					
Gene amplification	FISH	Amplified in 10%, high in 64% of cases (105)	ERα	ND	[6]
Gene amplification	SB	Amplified in 4.8% of cases (1157)	ERα, PR, MDM2 and FGFR1	Large tumor size	[34]
mRNA expression	FISH	High in 35% of DCIS (31), 31% of invasive tumors (83), 38% of metastases (21)	ERα, PR, p53 and HER-2	High tumor grade	[35]
Gene amplification/Protein expression	SB/IHC	Amplified in 1.6% of cases (124), nuclear staining in 16% of cases (115)	ERα	Successful hormonal therapy	[36]
Protein expression	WB	High in 46.5% of cases (316)	HER-2	Worse outcome with tamoxifen therapy	[37]
Gene amplification/Protein expression	FISH/IHC	Amplified in 5% of cases (362), high nuclear staining in <50% of cases (377)	ERα, HER-2	High relapse of HER1-3 positive cases with tamoxifen therapy	[38]
mRNA expression	qPCR	Upregulated in malignant tissue compared with normal tissue (64)	HER-2	The level increased under tamoxifen therapy and associated with poor outcome	[39]
Gene amplification/mRNA expression	qPCR	No amplification (127), high in 13% of cases (23)	ND	ND	[40]
**Ovarian cancer**					
Gene amplification	SB	Amplified in 7.4% of cases (122)	ND	ND	[34]
Gene amplification	FISH	Amplified in 25% of cases (24)	ERα	Poor overall survival	[41]
Protein expression	IHC	High in 68.7% of cases (83)	p53 and Bcl-2	ND	[42]
Protein expression	IHC	Higher in stage III and IV cases (471)	ERα, HER-2, PAX2, PAR6	Worse overall survival and poor outcome with carboplatin	[43]
Q region polymorphism	PCR	Short genotype in 40% of cases (89)	ND	Poor survival	[44]
**Endometrial cancer**					
Gene amplification/mRNA expression	qPCR	No amplification (30), high in 17% of cases (30)	ND	ND	[40]
mRNA expression	qPCR	High in 50% of cases (50)	ND	Poor overall survival	[45]
Protein expression	IHC	High in 93% of cases (82)	ERα	Poor prognosis	[46]
**Cervical Cancer**					
Protein expression	IHC	High in 52.7% of cases (108)	ND	Poor prognosis and outcome with CRT	[47]
Prostate cancer					
Protein expression	IHC	High in 80.6% of cases (36)	ND	High tumor grade and poor disease specific survival	[48]
Protein expression	IHC	High in about 50% of cases (480)	PSA recurrence	Poor overall survival	[49]
mRNA expression	qPCR	High in metastasis cases (58)	ND	Invasion and metastasis	[50]
**Thyroid cancer**					
Protein expression	IHC	High in 60.2% of primary tumors (83) and 73.5% of lymph node metastasis (46)	ND	High metastasis	[51]
Protein expression	IHC	Higher nuclear staining in ATCs than in normal thyroid tissues	Ki67	ND	[52]
**Hepatocellular carcinoma**					
Gene amplification	FISH	Amplified in 25% of total cases (311), 41% of metastatic cases (39) and 60% of recurrent tumors (15)	ND	Large tumor size and poor prognosis	[53]
Protein expression	IHC	High in 51.1% of cases (139)	Serum α-fetoprotein	Poor overall survival	[54]
Protein expression	WB	High in 68% of cases (34)	PCNA and MMP-9	SRC-3 postive HCC may be more invasive.	[55]
**Cholangiocarcinoma**					
Protein expression	WB	High in 55% of cases (20)	p-Akt and Bcl-2	ND	[56]
**Pancreatic adenocarcinoma**					
Gene amplification/Protein and mRNA expression	FISH/IHC	Amplified in 37% of cases (46), high protein in 64.5%, high mRNA in 73.7% of cases (78)	ND	ND	[57]
Protein expression	IHC	High in 68% of metastatic cases (28) and high in 44% of metastatic cases (48)	Low level of E-cadherin	ND	[58]
**Non-small-cell lung cancer**					
Gene amplification/Protein expression	FISH/IHC	Amplified in 8.2% of cases (134), high in 48.3% of cases (230)	ND	Poor disease specific survival	[59]
Protein expression	IHC	High in 27% of cases (311)	ND	Poor disease-free and overall survival, EGFR TKI resistance	[60]
**Colorectal carcinoma**					
Gene amplification/Protein expression	FISH/IHC	Amplified in 10% of cases (59), high in 35% of cases (85)	p53 and DNA aneuploid	Later clinical stages	[61]
**Gastric cancer**					
Gene amplification/mRNA expression	FISH/qPCR	Amplified in 7% of cases (72), high in 40% of cases (40)	ND	High tumor grade and poor prognosis	[62]
Gene amplification/mRNA expression/	FISH/qPCR	Amplified in 35.3% of cases (133), high in 70% of cases (30)	ND	Poor overall survival	[63]
Protein expression	IHC	High in 53.3% of cases (60)	p-Akt	Poor overall survival	[64]
**Bladder cancer**					
Gene amplification/Protein expression	FISH/IHC	Amplified in 7% of cases (71), high in 32.5% of cases (163)	Ki67	Poor prognosis	[65]
Protein expression	IHC	High in 31.5% of cases (146)	ND	High tumor grade and poor progression-free survival	[66]
Protein expression	IHC	High in 46.6% of cases (133)	ND	Poor survival	[67]
Protein expression	IHC	High in 46.8% of cases (109)	ND	Shorten recurrence interval	[68]
**Glioma**					
Gene amplification	qPCR	Amplified in 24.3% of cases (115)	ND	Poor survival in female and radiotherapy resistance	[69]
Gene amplification	qPCR	Amplified in 44.7% of cases (114)	HER-2 in male	Poor survival and radiotherapy resistance in female	[70]
**Nasopharyngeal carcinoma**					
Gene amplification/Protein expression	FISH/IHC	Amplified in 7% of cases (46), high in 51% of cases (71) and in 72% of cases with metastasis (25)	Ki67	Later clinical stages	[71]
**Esophageal squamous cell carcinoma**					
Gene amplification/Protein expression	FISH/IHC	Amplified in 13% of cases (115), high in 46% of cases (203)	Ki67	Later clinical stages	[72]
Protein expression	IHC	High in 64.3% of cases (98)	ND	Later clinical stages, CRT resistance and poor survival	[73]
Protein expression	IHC	High in 47.7% of cases (302)	ND	Later clinical stages, poor overall and progression free survival	[74]
**Bone cancer**					
Protein expression	IHC	High in 74.5% of cases (94)	ND	Age related differences in cartilage and giant cell tumors	[75]

n, number of cases; ND, not determined; FISH, fluorescence in situ hybridization; SB, Southern blot; IHC, immunohistochemistry; qPCR, quantitative PCR; WB, Western blot; PSA, prostate-specific antigen; CRT, chemoradiotherapy.
